# Efficacy of Environmental Sampling Devices for *Listeria monocytogenes* Detection in a Ready-to-Eat Production Facility

**DOI:** 10.3390/foods15081313

**Published:** 2026-04-10

**Authors:** David Tomás Fornés, Alba Fornés Pérez, José Manuel Barat Baviera, Yolanda Moreno Trigos, Ana Fuentes López

**Affiliations:** 1 Instituto Universitario de Ingeniería de Alimentos—FoodUPV, Universitat Politècnica de València, Camino de Vera s/n, 46022 Valencia, Spain; datofor@doctor.upv.es; 2 Instituto Universitario de Ingeniería del Agua y Medio Ambiente—IIAMA, Universitat Politècnica de València, Camino de Vera s/n, 46022 Valencia, Spain; alforpe@alumni.upv.es (A.F.P.); ymoren@upvnet.upv.es (Y.M.T.)

**Keywords:** *Listeria* spp., *Listeria monocytogenes*, surface sampling, environmental monitoring, biofilm formation, microbial contamination, test method, microbial genomics

## Abstract

*Listeria monocytogenes* is considered a highly persistent risk for public health in food production facilities. Food business operators manufacturing ready-to-eat foods (RTE) are required to sample processing areas for *L. monocytogenes* as part of their environmental monitoring plans. The aim of the study was to identify suitable sampling devices, demonstrating the crucial role of the sampling technique in the method performance for *L. monocytogenes* monitoring. Detection of *Listeria* spp. and *L. monocytogenes* from surfaces in an RTE food production facility was evaluated by using two different sampling methods (swabs and sponges). When using swabs, 46 sampling points were negative for both targets. However, when sampling same points with sponges, 30% samples (14 out of 46) were positive for *Listeria* spp. with 8 samples (17%) positive for *L. monocytogenes*. During subsequent in vitro experiments, *L. monocytogenes* strains spiked onto three different surfaces (stainless steel, Teflon and epoxy) showed recoveries of between 76% and 93% when using sponges, while in swabs, recoveries where always below 50%. All *L. monocytogenes* strains isolated belonged to the major clonal complexes (CC) circulating in Europe in food industry (e.g., CC121 and CC9) and none of them are considered among the hypervirulent strains. Genomic analysis, including new tools for source tracking (Gene Up Typer, bioMérieux) showed differences between strains isolated from different risk hygienic zones.

## 1. Introduction

*Listeria monocytogenes* is a relevant foodborne pathogen including special genotypic and phenotypic features that increase the risk of persistence in food production facilities, in particular for ready-to-eat products.

Over the last 2 years, *L. monocytogenes* infections have increased in the European Union with 5.8% more cases compared with the rate in 2022 (0.63 cases per 100,000 population), the highest rate and number of cases reported since 2007 with 335 deaths (19.7% case fatality). There have been numerous documented outbreaks of human listeriosis linked to meat and ready-to-eat products, with strong evidence of *L. monocytogenes* persistence in the associated food production environment [1]. In the context of other monitoring activities, the occurrence of *L. monocytogenes* indicates a reasonably foreseeable contamination rate in ‘ready-to-eat’ (RTE) food categories [2]. Growth at temperatures below 4 °C and the ability to generate biofilms and survive in environmental niches of food processing plants is a real threat for cross-contamination.

The risk for public health associated with *L. monocytogenes* in food environments for RTE products is specifically described in European regulation [3], where specific rules for testing and sampling are established. In this sense, food business operators manufacturing RTE foods are required to sample processing areas and equipment for *L. monocytogenes* as part of their sampling scheme. Similarly, implementation of effective environmental monitoring programmes is also addressed in FSMA by the Preventive Controls for Human Food (PCHF) [4].

In order to fulfil legal requirements, adequate cleaning and sanitizing of all food-contact surfaces is critical in preventing cross-contamination of foods by pathogens, especially in preparing RTE foods. However, pathogen recovery efficiency is highly dependent on the sampling tools used [5]. The efficacy of the detection methods can be impacted by the devices employed for microbiological environmental sampling on different surfaces. *L. monocytogenes* detection is highly affected by the sampling procedure [6,7], due to its capability of generating biofilms and strong attachment to the surfaces [8,9]. In this sense, the presence of genes associated with biofilm formation may explain the different attachment behaviour of bacteria and, consequently, the different recoveries obtained for the sampling methods [10,11].

In our study, we evaluated the recovery of *L. monocytogenes* in real RTE food environment conditions by using two different surface sampling methods (swabs and sponges with neutralizers). Efficacy of the recovery of isolated strains was also performed from different surfaces, confirming the results obtained in real conditions. Tools for source tracking and genetic characterization were applied to identify genetic profiles associated with virulence and biofilm formation, and the impact on the recovery of the strains by the applied sampling techniques.

## 2. Materials and Methods

### 2.1. Sampling Points in the Production Facilities

Sampling was performed in a RTE factory after cleaning and disinfection, and just before starting the production. The sampling points selected were part of the standard operation procedures already defined by the food company and included in the HACCP plan to check the effectiveness of the cleaning operations on a weekly basis. A total of 46 sampling points were identified. All of them were located in the high-hygiene area of the factory where the finished multicomponent RTE products (consisting of cooked meat, vegetables and cheese) were combined and packaged. All surfaces were sampled by one trained technician with more than 20 years of experience in surface sampling and microbiology. The sampling was performed first with one sampling technique (swab or sponge) and, immediately after, the same point and surface size was sampled on the surface next to it with the other method.

The sampling points included food-contact surfaces areas (n = 25, zone 1) and non-contact surface areas (n = 21) close to the product line (zone 2) or in the production area (zone 3) (Table 1). In total 22 samples were obtained from one packaging line (Line A) and 16 samples were taken from another packaging line (Line B), including conveyor belts, machine control panels, equipment, slicing/cutting parts, and Teflon protecting parts. The remaining samples (n = 8) were from common areas in the production area (e.g., handrails, doors, boxes).

For flat sampling points, an area of at least 100 cm^2^ (10 cm × 10 cm) was sampled by each sampling device. Most of the surfaces were non-flat areas and the maximum surface area available (at least 300 cm^2^) was sampled by each device. Exceptions were done when the maximum sampling area was lower than the values given before (e.g., automatic door clean room opening button).

### 2.2. Sampling Techniques

In the present study, two different sampling techniques were evaluated. The sampling protocol used by the RTE factory (swab), as standard operational procedure, consisted of the use of a cotton stick swab from sterile wrapping (DELTALAB, Barcelona, Spain) not containing any neutralizer or diluent. The tip of the swab was placed on the wet surface to be examined and streaked, while rotating the stick swab between thumb and forefinger. For flat surfaces, the sampling was performed horizontally and vertically (10 times in each direction). For other points, the same protocol was followed, sampling the entire wet surface, including crevices, gaps, connections, etc. After sampling, the humid stick swab was returned to the tube, transferred to the laboratory and submerged in Half Fraser broth (10 mL).

Alternative sampling protocol (sponge) consisted of the use of irradiated polyurethane sponges with a square size of 11.5 cm × 23 cm and 2.5 mm thickness (PolySponge™ World bioproducts LLC, Libertyville, IL, USA) in a plastic bag containing 16 mL HiCap™ neutraliser (Whirl-Pak^®^, Pleasant Prairie, WI, USA). Plastic bag containing the sponge and neutralizer agent was opened. Sponge was removed aseptically with sterile gloves. The surface was then sampled horizontally and vertically using even and firm pressure, changing the face of the sponge ensuring the whole area is sampled. The sponge was returned to the plastic bag with the neutralizer and remained moistened, transported to the laboratory and submerged in Half Fraser broth (100 mL).

### 2.3. Listeria Monocytogenes Isolation and Genomic Analysis

Samples were stored at a temperature between 1 °C and 8 °C and analyzed within 24 h of sampling. *Listeria* spp. and *L. monocytogenes* analyses were performed, regardless of the sampling procedure, using the standard protocol described by the International Organization for Standardization (ISO) [12]. Briefly, the sampling device was submerged in the enrichment broth (10 mL of Half Fraser broth (Scharlab S.L., Barcelona, Spain) directly added to the swab or 100 mL of Half Fraser broth added to the sponge and neutralizer) and incubated at 30 °C for 24 h. After incubation, enrichment broths were streaked on Agar Listeria Ottaviani Agosti and PALCAM agar (Scharlab S.L.) and incubated at 37 °C for 24–48 h. In parallel, secondary enrichment was performed in Fraser broth at 37 °C for additional 24 h and streaked again as described before. Typical isolated strains were confirmed as *L. monocytogenes* by biochemical tests (API Listeria, bioMérieux, Lyon, France). For *Listeria* spp. (non monocytogenes), confirmation was performed by microscopic observation and catalase test.

Molecular typing of the confirmed *L. monocytogenes* strains was performed using the Gene-Up^®^ Typer (bioMérieux, France) with its proprietary technology. Briefly, DNA was extracted by mechanical lysis from isolated strains. DNA suspension of each strain was then transferred to one PCR strip containing 2 accessory gene markers in each of the 8 wells resulting in a 16-marker absence/presence profile for each sample. Data models use this 16-marker profile to define clusters and predict clonal complexes in the sample, based on an algorithm developed from reference strains defined by a single-linkage core-genome MLST tree.

Finally, Whole Genome Sequencing (WGS) of four selected *L. monocytogenes* strains from different clusters was performed. For this purpose, DNA was extracted from each isolate using the GeneJET™ Genomic DNA Purification kit (ThermoScientific, Darmstadt, Germany) following the Gram-positive protocol. Sequencing and pre-processing of reads (trimming of adapters and quality control) was performed by Seqplexing S.L. (Paterna, Spain). Briefly, libraries were performed using the kit xGenDNA Library Prep EZ (IDT, Coralville, IA, USA) and verified by QIAxcel Advanced System (Qiagen, Hombrechtikon, Switzerland) followed by sequencing by paired-end (2 × 250 pb) using MiSeq sequencer (Illumina, San Diego, CA, USA).

After quality assessment with FastQC (https://www.bioinformatics.babraham.ac.uk/projects/fastqc/, accessed on 12 August 2023), data generated in Fastq file format was assembled using the SPAdes software v3.1. [13]. Species assignment was validated by Average Nucleotide Identity (ANI) analysis using FastANI v0.1.3 [14]. Contigs were compared against the *L. monocytogenes* strains EGD-e, F2365, 10403 and 6179. Additionally, *L. innocua* CLIP 11262 was added as an outgroup. Reference genomes were obtained from GeneBank. An ANI value >95% was considered as evidence of conspecificity [15].

Gene annotation was performed with Prokka using the databases through the modules Prodigal and Aragorn [16]. Biofilm-producing genes were selected from references in the literature [10,11,17,18], and aligned against the assemblies from each strain using BLAST (Version. 2.17.0, cut-off of >95% of nucleotide identity and e-value < 10^−6^). Antimicrobial resistant (AMR) and virulence factor (VF) genes were detected using ABRicate (https://github.com/tseemann/abricate, accessed on 12 August 2023) against MEGARes [19] and VFDB [20] databases, respectively. For mobile genetic elements (MGEs), MobileElementFinder [21] was used. Furthermore, plasmid presence was assessed using PlasmidFinder [22]. Heatmap construction was performed using the tidyverse and ggplot2 packages in R (v4.5.2).

### 2.4. Performance for Sampling Techniques on Artificially Contaminated Surfaces

In order to estimate the efficacy of the sampling devices tested in industrial conditions, additional experiments were performed in controlled laboratory conditions. Three different surfaces were selected: stainless steel (A-316), Teflon (PTFE or Politetrafluoroetilen) and epoxy. Surfaces of 100 cm^2^ each were disinfected with alcohol and UV light prior to being contaminated.

Two *L. monocytogenes* strains isolated from different origins, belonging to different clusters and clonal complexes (CC), were selected based on the different genetic profiles and isolation environmental hygienic conditions. Strain #32 isolated from a food-contact surfaces from conveyor belt (zone 1, CC 5) and #117 from a drain located in the production area (zone 3, CC 9)) were grown in Brain Heart Infusion (BHI, Scharlab S.L.) overnight and diluted tp a concentration of around 4 log_10_ cfu/mL in Buffered Peptone Water (BPW, Scharlab S.L.) with albumin (3 g/L) to simulate dirty conditions. *L. monocytogenes* suspensions from each strain were artificially contaminated in triplicate on each surface (100 cm^2^) by applying 25 droplets of 20 µL each. This protocol was repeated twice (to perform sampling with both devices). After contamination, surfaces with the inoculum were dried into a flow cabinet for 1 h and then stored in a sterile box at room temperature for 24 h.

From each spiked surface, sampling procedures with swabs and sponges were performed using the same protocol applied in the food facilities, as described above. After sampling, swabs and sponges were submerged in BPW (10 mL and 100 mL, respectively) and homogenized. Suspensions were spread on Trypticase soy agar (TSA, Scharlab S.L.), incubated at 37 °C for 24 h and the results from the colony counts were expressed in cfu/100 cm^2^. Percentage of recovery was estimated dividing the counts obtained for each condition by the spiked concentration (both in log_10_ cfu/100 cm^2^). When positive colonies were detected but in low numbers (1, 2 or 3) results were expressed as detected but not quantified, according to ISO 7218:2025 [23]. In these cases, the respective limit of quantification (LOQ) is expressed.

### 2.5. Statistical Analysis

The statistical program used for data processing was Statgraphics Centurion XIX (The Plains, VA, USA). A two-way ANOVA was employed to test for significant differences in Listeria counts across sampling devices (swab and sponge) for each surface material (stainless steel, Teflon, and epoxy) and their interaction. The post hoc LSD (least significant difference) procedure was applied to identify differences that were set at *p* < 0.05.

## 3. Results

### 3.1. Isolation of Listeria spp. and L. monocytogenes in the Production Facilities with Different Sampling Devices

When using the swab sampling device, all 46 samples were negative for *Listeria* spp. and *L. monocytogenes* detection. However, when using the sponge device with neutralizer for sampling, 30% samples (14 out of 46) were positive for *Listeria* spp., with 8 samples (17% from the total) being positive for *L. monocytogenes* (Table 1).

Differences between sampling devices were statistically significant at 99% confidence level (*p* ≤ 0.01). Five out of eight positive results for *L. monocytogenes* were detected in food-contact areas. These strains were isolated from the conveyor belts (#32 and #36), 1 strain from the PVC strip curtain (#47) on the packaging line A, and 2 strains from 2 sampling points on the vibrational platforms from both packaging lines (#42 and #54). The 3 positives from non-contact zones were detected in common areas from the clean room (button to open access door (#2), handrail (#9), and drain (#117)). Six additional surfaces showed the presence of *Listeria* spp. other than *L. monocytogenes*, mostly from packaging line A. From these, 5 were isolated from food-contact surfaces: 3 strains in the conveyor belt surfaces line A (#29, #31, and #37), 1 in the conveyor belt from line B (#53), and 1 in the guidance cutting system line A (#38). Only one stain was isolated from one non-contact surface: roller packaging film line A (#35).

**Figure 1:**
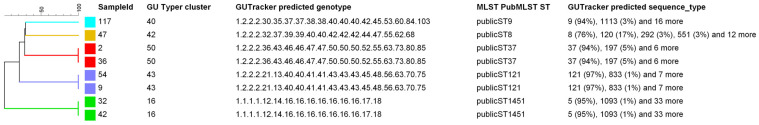
Clustering showing genetic relationship between the strains of *L. monocytogenes*.

Molecular typing performed for *L. monocytogenes* by cgMLST and Gene-Up Typer systems showed equivalent results with a total of five clusters (Figure 1). One cluster included 2 isolates from food-contact surfaces of the same line A (#32 and #42) and other cluster included only one isolate from same line (#47). Each of the other 2 clusters included two surfaces from both food-contact (#36 and #2) and non-contact surfaces (#54 and #9) belonging to the 2 packaging lines and common areas. Another cluster included only one surface from a drain (#117).

Clonal complex (CC) predicted by Gene Up Typer showed each cluster belonging to a different group: CC37 for #2 and #36; CC5 for #32 and #42; CC121 for #54 and #9; CC8 for #47 and CC9 for #117. Finally, whole genome sequencing (WGS) of four selected strains was performed to investigate the presence of disinfectant resistance genes, biofilm-associated genes and virulence factor genes (VFG). Firstly, ANI values confirmed the classification of all isolates as *L. monocytogenes* (>95% identity) and their divergence from *L. innocua* (<90%). Notably, while isolates #117, #2, and #54 showed high affinity to Lineage II references (EGD-e, 6179), isolate #32 clustered with the Lineage I strain F2365 (99.4%).

**Figure 2:**
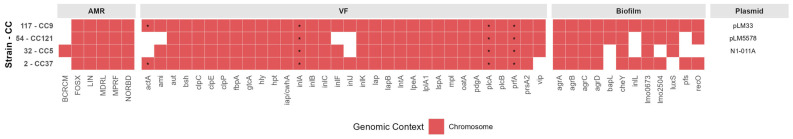
Antimicrobial resistance (AMR), virulence factors (VF), biofilm-associated (Biofilm) genes and plasmids (Plasmid) identified in four *Listeria monocytogenes* strains. Heatmap cells indicate chromosomic location of the gene (Chromosome). White cells indicate the absence of the gene. Asterisk (*) marks VF genes also associated with biofilm formation.

The presence of plasmids pLM33, pLM5578 and N1-011A was observed in strains #32, #54 and #117, respectively, whereas strain #2 was found to be plasmid-free. As demonstrated in Figure 2, the analysis revealed the presence of AMR and VFG genes in all strains examined. Notably, no association was observed between these genes and mobile genetic elements (MGEs) or plasmids, suggesting that these resistance and virulence determinants are encoded within the chromosome. Furthermore, most of the selected biofilm-associated genes were detected in all strains.

Alignment of genome sequences obtained from strains showed high homology with some specific genes associated with biofilm production in *L. monocytogenes* (Table 2) and for antimicrobial and stress resistance (Table 3).

### 3.2. Performance of Sampling Devices in Different Artificially Contaminated Surface Materials

In vitro tests were carried out using three different surface types in order to evaluate the recovery of *L. monocytogenes* from each surface, depending on the sampling system employed. For this purpose, 2 different strains isolated from food-contact surfaces (#32) and non-food-contact surfaces (#117), were employed (Table 4).

*L. monocytogenes* strain (#32) was spiked (4.9 log_10_ cfu/100 cm^2^) on to stainless steel, Teflon (PTFE/Politetrafluoroetilene) and epoxy surfaces and then sampled with a swab and sponge. The sponge with neutralizers provided *L. monocytogenes* average values of 3.93 ± 0.28 log_10_ cfu/100 cm^2^; 4.08 ± 0.40 log_10_ cfu/100 cm^2^ and 3.71 ± 0.48 log_10_ cfu/100 cm^2^, stainless steel, Teflon and epoxy surfaces (80%, 83% and 76% of recovery, respectively). Samples tested with the cotton swab, *L. monocytogenes* was detected in all surfaces but in a lower concentration, below 2 log_10_ cfu/100 cm^2^.

Same study performed with *L. monocytogenes* strain (#117) spiked at 4.0 log_10_ cfu/100 cm^2^ provided average values of 3.69 ± 0.11 log_10_ cfu/100 cm^2^, 3.15 ± 0.16 log_10_ cfu/100 cm^2^ (93% and 79% recovery) when using sponges on stainless steel and Teflon. In epoxy resin, *L. monocytogenes* was detected but with non-quantifiable concentrations (<2 log_10_ cfu/100 cm^2^). With cotton swabs, recovery results of strain #32 were similar to the previous strain (#117), with *L. monocytogenes* detected with <2 log_10_ cfu/100 cm^2^ and not detected in stainless steel.

A two-way ANOVA was applied to assess differences between groups (sampling device and surface material) and their interaction. The results are shown in Supplementary Table S1.

## 4. Discussion

### 4.1. Impact of Sampling Techniques in Detection of Listeria spp. and L. monocytogenes in Real Industrial Environment

The sampling techniques applied for *Listeria* detection in the RTE factory showed significant differences (95%), indicating the relevance of the sampling devices and diluents to the results obtained. In our study, sponges embedded in neutralizer broth showed a total of 31% positives for *Listeria* spp. and 19% positive results for *L. monocytogenes* in ready-to-eat meat-based hygienic zones and equipment, while no positive results were detected for both targets when using cotton swabs without neutralizer. Our results, when sampling with sponges, were similar to the results obtained in three Belgian pork cutting plants [24], showing 13% of environmental samples tested positive for *L. monocytogenes*, and are also similar to the 10% of recovery obtained in similar conditions in dairy industry by other authors [25].

Considering as a whole the values obtained in real factory conditions and in different strains and surfaces tested in the laboratory, we demonstrated that cotton swabs are not a suitable device to perform pathogen sampling for *Listeria* spp. and *L. monocytogenes*. In this sense, Keeratipibul et al. [26] also observed, when sampling wet surfaces, that cellulose sponge swabs had the highest recovery efficiency (94.4%) compared with cotton swabs (84%). When sampling dry surfaces, all swab materials showed significantly reduced efficiency, with cotton swabs achieving only 48.5%. Those results are similar to the values obtained in this study with sponges on dry surfaces (76–93%); however, in our study, cotton swabs showed a much lower recovery. In our study, the results are limited to cotton swabs. It should be considered swabs from different materials may have a relevant impact on bacterial recovery, including foam swabs, calcium alginate, Dacron^®^ and plastic [27]. Maes et al. [28] also showed more positive samples with biofilm contamination when using sponge stick after the cleaning and disinfection when compared with swab method. El-Moghazy et al. [29] reported low recovery for traditional cotton swabs (20–60%) when sampling stainless steel material, but still higher than the data obtained in our study, probably because the strains recovered in our study showed greater adherence to the surfaces tested. These results could indicate that neutralizer agents may also play a crucial role in the recovery from industrial conditions [30,31,32].

*L. monocytogenes* detection in food-contact surfaces when using sponges (5 out of 25: 20%) was higher than in non-food-contact surfaces (3 out of 21: 14%) indicating a high risk for cross-contamination of finished RTE products. The swabbing technique was clearly not able to identify the presence of *L. monocytogenes* in the factory, and consequently, preventive actions to avoid the risk of cross-contamination in finished products would not have been implemented.

In previous studies, it was indicated that compact cotton swabs can absorb more fluid thanks to natural cellulose fibres with high absorbency and bacteria can be tightly wrapped around the shaft [33,34], having lower performance because their compacted structure does not allow the release of the microorganisms in the diluent [35,36]. However, in our study, bacteria grew directly in the sampling device submerged in enrichment broth and no detachment was required to obtain final positive results. We can associate the lack of recovery to the inability of the cotton swabs to detach the microorganisms from the surface instead of to the lack of release in the diluent.

Summarizing, the results observed in the literature are aligned with the values obtained with sponges in our study, while the negative results obtained with cotton swabs indicate their lack of recovery and the limitations of these devices.

### 4.2. Performance of Sampling Devices to Recover Listeria spp. and L. monocytogenes in Spiked Surfaces

The in vitro tests performed on three different materials (stainless steel, Teflon and epoxy) with the selected *L. monocytogenes* strains confirmed that cotton swab performance was extremely low compared with sponges. Sponge recovery values ranged from 76% to 93% across the three different surfaces, while swabs recovered less than 50% of the total spiked population in all cases. Despite the relatively high concentration of *L. monocytogenes* spiked (4 log_10_ cfu/cm^2^), swabs provided negative or non-quantifiable levels, not allowing comparison between material surfaces. The low recovery from swabs (<50%) for *L. monocytogenes* is consistent with the data reported by Salo et al. [37] for other bacteria (*E. coli*, *B. cereus*, and *E. cloacae*), which obtained recoveries of 20.2–30.4% from stainless steel. Branck et al. [8] also demonstrated recoveries lower than 30% when sampling biofilms with swabs. In addition, Kovačević et al. [7] showed that the cotton swab method was significantly (*p* < 0.01) less efficient in the recovery of *Listeria* spp. than the sterile sponge.

Previous studies on the ability to detect *L. monocytogenes* on surfaces also showed no or small significant effects of the surface type [38,39,40]. In our study, when sampling with sponges, we found no significant differences (*p* > 0.05) between the three materials evaluated for one of the strains tested (#32, isolated from food-contact surfaces) (Table S1). However, when evaluating the other strain recovered from a different environmental condition (#117, drain), recovery from stainless steel and Teflon was equivalent, whereas recovery from epoxy was significantly lower (*p* < 0.001) than the other two materials (Table S1).

Although all strains may have the ability to adhere to different surfaces [9,41], the specific characteristics of the strain isolated from the drain (#117), in particular, the resistance traits in their natural environment and their genetic profile, may play a role in the attachment and recovery, reducing the probability of recovery or even producing negative results depending on the device for sampling.

Genetic profile from strain #32, isolated from contact surface and showing good and equivalent recovery rates in all materials, does not contain some genes associated with biofilm formation in food-contact surfaces and adhesion, like inlL, lmo2504, prfA and bapL [11,18]. All these genes were present in strain #117 with almost 100% homology. This may suggest that the genetic profile of the strain and the origin of the strain [42] can be associated with the better adhesion and biofilm formation to the polymer material, with a potential impact on false negative results due to the lower recovery in epoxy surfaces. More strains with a similar genetic profile and isolation sources would contribute to confirming this assumption.

The better recovery obtained with sponges compared with swabs, indicates the sampling device and use of suitable neutralizer is playing a crucial role in the estimation of the bacterial cleaning and disinfection protocols, increasing the risk of false negatives and generating incorrect conclusions when evaluating the effectivity of sanitation operations.

### 4.3. Risk Associated with L. monocytogenes Isolates

All strains isolated in the present study were considered within the major clonal complexes (CC) circulating in the European food industries with different prevalences, and none of them are considered among the hypervirulent strains [43]. Both CC121 and CC9 detected in this study in two and one strain, respectively, are the most frequent clonal complexes isolated from food environments and considered food-associated clones, showing limited virulence and rarely associated with human clinical cases. Other clonal complexes detected in this study which are considered intermediate clones, such as CC8, CC5 and CC37, are reported in both food and clinical isolates [44]. All the strains, except #32 and #42 (more distant from the other isolates and both from very close-related food-contact surfaces from the same packaging line), belong to the same lineage II, indicating a potential high dispersion of the strains from the same origin in the factory. Indeed, isolates detected in the same facilities during production were closely related to the strains presented in this study, demonstrating the importance of the dispersion of the strains associated with deficiencies during cleaning and disinfection steps. These results reinforce the impact of the sampling technique and the ability of the sponge sampling device to recover the most important strains isolated, including food but also clinical related CCs.

Despite the genetic diversity among the analyzed CCs, the antimicrobial resistance profile remained remarkably stable. All isolates shared a conserved genetic backbone comprising fosX, lin, norB, mdrL, and mprF. The first three have been identified as intrinsic resistance genes in most *L. monocytogenes* strains [45]. These genes confer protection against antibiotics (fosfomycin, lincosamides, fluoroquinolones, and macrolides) and facilitate host immune evasion through mechanisms such as enzymatic modification (fosX) [46], multidrug efflux pumps (mdrL, norB) [47], and alterations in cell envelope charge (mprF) [48]. This underscores the preservation of these chromosomal genes within the species.

The sole variability observed was the distinct presence of the bcrCM gene in strain #32. This gene encodes an efflux pump that extrudes antibiotics and disinfectants from the cell, conferring multidrug resistance (MDR) to the bacteria [49]. Since no association with MGEs was detected for any of these genes, the presence of bcrCM in a single isolate highlights a specific chromosomal divergence in the CC5 lineage compared to the otherwise identical resistomes of the CC9, CC37, and CC121 strains. Similarly, VFs were highly conserved across the studied lineages, albeit with specific divergences (Figure 2). Therefore, *Listeria* Pathogenicity Island 1 (LIPI-1), was present in all isolates, governed by the master regulator prfA. This gene is indispensable for orchestrating the expression of the virulence regulon, a process strictly dependent on environmental cues such as high temperature and stress [50]. Central to this island is hly (encoding listeriolysin O), a gene responsible for pore formation to facilitate pathogen entry. Since this gene is exclusive to pathogenic *Listeria* spp., it serves as a critical diagnostic marker for assessing virulence in isolates from RTE foods [51]. Alongside hly, the island includes plcA and the operon mpl-actA-plcB, which are essential for phagosome escape and cytosolic motility [52,53]. Furthermore, a core set of adhesion and invasion genes was ubiquitous. Notably, inlA was identified in all strains; this internalin plays a pivotal role in the initial adherence to host intestinal cells, marking a critical step in the infection process [54]. As obtained in our study, Parra-Flores et al. [55] detected hlyA, prfA and inlA in silico and in vitro in RTE isolates.

While the CC9 strain (#117) exhibited the complete set of VFs, notable variability was observed in the accessory virulence profile of the other lineages. Remarkably, strain #54 (CC121) and #32 (CC5) presented absence of the actA gene in the typically conserved operon. Additionally, internalin inlJ was not detected in both strains. In addition, the autolysin amidase ami and the internalin inlF were absent in the strain #54, which displayed the most characteristic profile. On the other hand, the surface adhesin vip was absent in the strain #2 (CC37). This mosaic distribution suggests that while the central pathogenic machinery is preserved, lineage-specific losses may modulate the precise virulence strategies of each CC.

Regarding plasmid presence, N1-011A plasmid has been previously detected in CC5 isolates in RTE processing plants [55], whereas pLM5578 plasmid was detected in food chain facilities [17]. Both of them have been associated with resistance to disinfectants like benzalkonium chloride [17,20]. In addition, pLM33 confers tolerance to processing-related stresses—such as low pH, high salinity, and oxidative stress—and enhances virulence [20].

Representative isolates from the clusters obtained showed a high proportion of genes involved in biofilm formation. In particular, the strain #117, isolated from a drain in the high-hygienic area, contained the highest homology with 16 genes identified in the literature (Table 2). The combination of a higher proportion of stress tolerance genes and the presence in the genome of inlL class 1 internalin associated with biofilm formation potentially involved in mucin binding, sessile growth, and adhesion. These characteristics may enable this isolate to persist in the food processing environment [11]. In addition, previous studies indicated that genes inlA, inlL, prfA, plcA, actA, Imo0673, bapL, recO, Imo2504, and luxS play an important role in biofilm formation on food-contact surfaces, such as stainless steel, aluminum, polycarbonate, polypropylene, polyurethane, polyvinylchloride, silicone rubber, natural white rubber, PETG, PTFE, Lexan, nitryl rubber, and glass [17,18,56].

Interestingly, in the two *L. monocytogenes* strains isolated from food-contact surfaces, where cleaning and disinfection are more intensive and accessible, the inlL gene was not present, indicating that these strains may be transient in the food environment and exhibit lower persistence and surface attachment. The strain isolated from a general non-contact food surface, such as the strain #2 (isolated from the button to open the door), showed an intermediate genetic profile, with the presence of inlL and the absence of bapL and luxS.

## 5. Conclusions

Sampling devices used for environmental monitoring play a crucial role in evaluating surface contamination by *Listeria* spp. and *Listeria monocytogenes*. Use of sponges with a suitable neutralizer allowed recovery of both targets in real conditions and in vitro tests, compared with the negative results obtained with cotton swabs.

Based on our results, with the limited information collected in the industrial environment, we recommend limiting the use of cotton swabs and applying this device only for sampling hard-to-reach areas that are inaccessible to scraper sponges, wipes and other sampling devices. Swabs with other material than cotton may also improve recovery in those cases, but this would need to be verified by a similar sampling exercise.

Using sponges with neutralizers instead of swabs should be considered as much as possible, aligned with recommendations from USDA and ISO guidelines. Genetic profiles obtained from isolates are related to bacterial attachment to specific surfaces, making isolation and detection more difficult.

## Figures and Tables

**Table 1:** Results from sampling points in the different hygienic zones.

Code	Description	Zone ^(1)^	Swab		Sponge	
			List. ^(2)^	L.mono ^(2)^	List. ^(2)^	L.mono ^(2)^
29	Horizontal surface conveyor belt. Line A	1	NEG	NEG	**POS**	NEG
30	Vertical surface horizontal conveyor belt. Line A	1	NEG	NEG	NEG	NEG
31	Vertical surface conveyor belt elevator. Line A.	1	NEG	NEG	**POS**	NEG
32	Conveyor belt after freezer tunnel. Line A.	1	NEG	NEG	**POS**	**POS**
33	Dispensers for nozzles. Line A	1	NEG	NEG	NEG	NEG
34	Dispensers for blades. Line A	1	NEG	NEG	NEG	NEG
36	Conveyor belt after heating system. Line A	1	NEG	NEG	**POS**	**POS**
37	Conveyor belt cooling system. Line A	1	NEG	NEG	**POS**	NEG
38	Guidance for cutting system. Line A	1	NEG	NEG	**POS**	NEG
39	Blades for cutting system. Line A	1	NEG	NEG	NEG	NEG
40	Cutting system. Packaging line A.	1	NEG	NEG	NEG	NEG
41	Conveyor belt after cutting system. Line A	1	NEG	NEG	NEG	NEG
42	Surface vibration system. Line A	1	NEG	NEG	**POS**	**POS**
43	Surface conveyor belt elevator Line A.	1	NEG	NEG	NEG	NEG
45	Distributor dosing system. Line A	1	NEG	NEG	NEG	NEG
47	PVC strip curtain after cooling system. Line A	1	NEG	NEG	**POS**	**POS**
10	Teflon surface distribution platform. Line B	1	NEG	NEG	NEG	NEG
50	Horizontal surface conveyor belt. Line B	1	NEG	NEG	NEG	NEG
51	Blades for cutting system. Line B	1	NEG	NEG	NEG	NEG
52	Guidance for cutting system. Line B	1	NEG	NEG	NEG	NEG
53	Conveyor belt after cutting system. Line B	1	NEG	NEG	**POS**	NEG
54	Surface vibration system. Line B	1	NEG	NEG	**POS**	**POS**
55	Surface conveyor belt elevator. Line B	1	NEG	NEG	NEG	NEG
57	Distributor dosing system. Line B	1	NEG	NEG	NEG	NEG
59	Surface cutting system. Line B	1	NEG	NEG	NEG	NEG
6	Button to control vibrational system. Line A	2	NEG	NEG	NEG	NEG
35	Rollers packaging film. Line A	2	NEG	NEG	**POS**	NEG
44	Weight control dosing system. Line A	2	NEG	NEG	NEG	NEG
46	Weight system. Line A	2	NEG	NEG	NEG	NEG
48	Rollers conveyor belt. Line A	2	NEG	NEG	NEG	NEG
49	Scrapper blade. Line A	2	NEG	NEG	NEG	NEG
1	Screen Line B	2	NEG	NEG	NEG	NEG
56	Weight control dosing system. Line B	2	NEG	NEG	NEG	NEG
60	Rollers conveyor belt. Line B	2	NEG	NEG	NEG	NEG
61	Scrapper blade. Line B	2	NEG	NEG	NEG	NEG
4	Handrail mobile ladder	2	NEG	NEG	NEG	NEG
8	Handrail platform for cooling system	2	NEG	NEG	NEG	NEG
9	Handrail ladder to food distribution platform	2	NEG	NEG	**POS**	**POS**
11	Box for rejected raw material	2	NEG	NEG	NEG	NEG
27	Rollers packaging film (backup)	2	NEG	NEG	NEG	NEG
28	Rollers printing packaging film (backup)	2	NEG	NEG	NEG	NEG
62	Bowls. Line B	3	NEG	NEG	NEG	NEG
5	Shovels removing residues. Line B	3	NEG	NEG	NEG	NEG
2	Automatic door clean room opening button	3	NEG	NEG	**POS**	**POS**
3	Shovels collecting residues	3	NEG	NEG	NEG	NEG
117	Drain close to Line B	3	NEG	NEG	**POS**	**POS**

^(1)^ Hygienic zones are defined as 1: food-contact surfaces; 2: Non-food-contact surfaces close to the production line; 3: Other non-food-contact surfaces in the production area. ^(2)^ List. = *Listeria* spp.; L.mono = *Listeria monocytogenes*. NEG: Not detected/surface; POS: Detected/surface.

**Table 2:** Alignment ratio for biofilm-related genes detected in the environmental isolates.

Genes	#2 (CC 37)	#32 (CC 5)	#54 (CC 121)	#117 (CC9)
acta	99.12	95.69	96.70	99.95
agrA	100	98.90	100	100
agrB	99.84	98.70	99.84	100
agrC	99.85	97.30	98.38	100
agrD	100	98.77	100	100
cheY	99.72	96.67	100	100
inlA	98.21	96.46	97.80	99.96
inlL	99.15	--	--	99.95
lmo0673	99.52	98.10	100	100
lmo2504	99.39	--	99.70	100
Pfs	99.86	--	--	100
plcA	98.22	97.48	98.64	100
prfA	98.24	--	98.24	100
recO	100	--	99.22	100
bapL	--	--	98.03	99.98
luxS	--	95.73	100	100

**Table 3:** Alignment ratio for antimicrobial and stress-resistance-related genes detected in the environmental isolates.

Genes	#2 (CC 37)	#32 (CC 5)	#54 (CC 121)	#117 (CC9)
fosX	98.5	100	94.74	100
vga(G)	98.09	98.09	98.09	100
bcrC	--	100	--	--
bcrB	--	100	--	--
qacH	--	--	90.98	--
cadC	--	--	100	100

**Table 4:** Microbial counts (log_10_ cfu/100 cm^2^) and recovery (%) of *L. monocytogenes* in different surfaces (stainless steel, Teflon, and Epoxy) using different sampling devices (sponge and swab) at 2 spiked levels (4.9 and 4.0 log_10_ cfu/100 cm^2^).

Sampling Device	Strain	Spiked Level	Stainless Steel	Teflon	Epoxy
Sponge	#32	4.9	3.93 ± 0.28 (80%)	4.08 ± 0.40 (83%)	3.71 ± 0.48 (76%)
Sponge	#117	4.0	3.69 ± 0.11 (93%)	3.15 ± 0.16 (79%)	Detected *
Swab	#32	4.9	Detected *	Detected *	Detected *
Swab	#117	4.0	Not detected ^†^	Detected ^†^	Detected ^†^

* Detected results are reported when a non-quantified number of colonies are obtained (below limit of quantification = 1.6). ^†^ Not detected results are <1.0 values.

## Data Availability

The original contributions presented in this study are included in the article/Supplementary Materials. Further inquiries can be directed to the corresponding authors.

## Supplementary Materials

The following supporting information can be downloaded at https://www.mdpi.com/article/10.3390/foods15081313/s1, Table S1: Results of Two-Way ANOVA assessing the effects of sampling device (SD) and surface material (SM) on Listeria counts.

## Abbreviations

The following abbreviations are used in this manuscript:

RTE	Ready-to-Eat
FSMA	Food Safety Modernization Act
HACCP	Hazard Analysis Critical Control Points
CC	Clonal Complex
